# Satisfaction level in dental-phobic patients with implant-supported rehabilitation performed under general anaesthesia: a prospective study

**DOI:** 10.1186/s12903-018-0644-x

**Published:** 2018-11-01

**Authors:** Louise Sidenö, Rim Hmaidouch, Jan Brandt, Nadine von Krockow, Paul Weigl

**Affiliations:** 1 Department of Postgraduate Education, Master of Oral Implantology, Oral and Dental Medicine at Johann Wolfgang Goethe-University, Theodor-Stern-Kai 7 / building 29, 60596 Frankfurt am Main, Germany; 2 Stockholm, Sweden; 3 Department of Dental Prosthodontics, Faculty of Oral and Dental Medicine at Johann Wolfgang Goethe-University, Theodor-Stern-Kai 7 / building 29, 60596 Frankfurt am Main, Germany; 4 Department of Oral Surgery, Faculty of Oral and Dental Medicine at Johann Wolfgang Goethe-University, Theodor-Stern-Kai 7 / building 29, 60596 Frankfurt am Main, Germany

**Keywords:** Patient satisfaction, Dental phobia, Pharyngeal reflex, Dental implants, Prosthetic rehabilitation, general anaesthesia

## Abstract

**Background:**

Phobic patients avoid dental treatment impairing their oral health and making it challenging to offer them prosthetic rehabilitation. This study evaluated patients’ experience of implant-supported prosthetic treatment after implantation performed under general anaesthesia due to dental phobia and severe pharyngeal reflexes (SPR). The effect of gender, age and location of implantation on patient satisfaction was tested.

**Methods:**

Two hundred five patients underwent implantation under general anesthesia both in maxilla and mandible, respectively. After a trans-gingival healing period of 6–8 weeks, fixed implant bridges were inserted. Patients completed oral health impact profile questionnaire (OHIP-14). An additional set of six special questions was also developed and considered. Analysis of the OHIP-14 total score was made using logistics regression. Wald chi-square test was used to analyse the effect of age, gender and location of implantation. Effect sizes were estimated as odds-ratios and associated 95% Wald confidence intervals.

**Results:**

Eighty two of 205 patients were included after prosthetic treatment. After start, 38 patients were excluded (4 died and 34 couldn’t be reached). OHIP-14-analyses were made by 43 patients (30–90 years). 67% of patients were totally satisfied with the whole implant rehabilitation (scoring 0). Mean of total score was 2.5. Only age affected significantly (*p* = 0.014) patients satisfaction. The obtained data indicate that younger patients (30–64 years) especially women are less satisfied (4.95) than older patients (0.3) for age group (65–90 years).Special questions’ data showed that 94.5% were satisfied with their treatment. 77.3% continued regular check-up after treatment and 96.9% would undergo the same treatment again. 95.5% would recommend implants to a friend of colleague.

**Conclusion:**

Gender and location of implantation have no significant influence on patient satisfaction. Younger patients especially women are less satisfied than older patients. Phobic patients are totally satisfied with implant rehabilitation under general anaesthesia which means that this treatment can be considered as a treatment of choice giving these patients the same opportunity like others to improve their oral health and well-being.

## Introduction

Anxious patients due to dental phobia or severe pharyngeal reflexes (SPR) show poorer oral health and more decayed and missing teeth than typical individuals [1]. Prosthetic treatments are needed for recovery of missing teeth in these patients, however, these patients are uncooperative and show poor dental treatment compliance which complicates any treatment; increases risk of failure and makes it difficult to perform implant-supported rehabilitation [2, 3]. A very long procedure is expected if implantation is considered for these patients. Consequently, local anesthesia will be insufficient to perform an adequate operation [4, 5]. In such cases, surgery under general anesthesia could be an option that enables patients undergoing implant treatment to improve their oral health, and well-being.

General anaesthesia makes it convenient for patients to have all surgical procedures carried out in one session and then implants can be installed in the maxilla or mandible or if needed in both jaws in one appointment [6]. As known, rehabilitation with implants prevents continuous alveolar bone resorption, preserves ridge height and width which ensures positive aesthetic outcome [7, 8], comfort and efficacy of prosthetic reconstruction [9–11]. Additional positive factors for patients are increase in self-esteem, and patients’ satisfaction [12, 13].

When assessing the outcome of implant treatment, it is important to consider both the clinicians’ and the patients’ appraisals [14–16]. For the clinicians, implant survival, prosthesis longevity, and the complications are the most important factors. On the other hand, cost effectiveness benefit, social and psychological impact of the treatment are more important for the patients [17, 18]. Patients’ satisfaction depends on function, comfort, esthetics and speech disruption [15, 17] and may represent a crucial factor of implant success for the patient [19–22]. Patient satisfaction is seen a vital aspect by evaluating the overall quality of dental rehabilitation and should be made on a regular basis to allow clinical practitioners to assess their services [23–25].

The Oral Health Impact Profile (OHIP) questionnaire is an instrument developed to be used in clinical studies [26–33] to measure Oral Health Quality of Life (OHRQoL). Several short versions of this tool have been developed, such as the version OHIP-14 which consists of seven subgroups with two questions for each one [27, 28, 31].

Most dental satisfaction studies were performed on general dental treatment [34] and patients with dental anxiety have been shown to be significantly associated with greater dissatisfaction [35]. However, no studies have addressed patients’ satisfaction suffering from dental phobia and SPR after implantation under general anesthesia; the perception of treatment outcomes by those patients is still missing. The aim of the study is therefore to evaluate satisfaction of partially edentulous patients suffering from dental phobia and SPR with their implant rehabilitation carried out under general anesthesia in one or both jaws. The effect of gender, age and location of implantation will be tested. This study evaluated patients’ experience of oral surgical and prosthetic procedures as well as their satisfaction with treatment outcome. The hypotheses of this study are:

- Patients suffering from dental phobia and SPR will experience good patient satisfaction after implant treatment under general anesthesia.
- age, gender and location of implantation will affect patients satisfaction.
- success of rehabilitation with implant fixed bridges by these patients is similar to that by patients treated without general anesthesia.

## Methods

The OHIP-14 questionnaire was used to measure patient satisfaction in this investigation. It is a 14-questions survey, grouped as seven domains: functional limitation, physical pain, psychological discomfort, physical disability; psychological disability, social disability, and handicap (Annex). The OHIP-14 has been previously translated into Swedish and the reliability and validity has been tested and recommended for use in studies in the Swedish population [28]. Additionally, a set of six special questions related to patients’ dental behaviour and treatment satisfaction (Table 1) was developed in Swedish and used as well. The study proposal was submitted to the ethical committee of Stockholm in Sweden (No 2014/1811–31/1). The board of the ethical committee did not see any ethical research obstacles to this study.

**Table 1:** Response frequencies for special questions

Question	Response	No. (%)
Are you attending regular check-up at Dentist/Hygienist?	Yes No	34 (77.3) 10 (22.7)
Are you satisfied with your implant bridges?	Yes No	42 (94.5) 2 (5.5)
Would you recommend implants to friend or colleague?	Yes No Don’t know	42 (95.5) 1 (2.3) 1 (2.3)
Would you recommend narcosis clinic?	Yes No Don’t know	41 (93.2) 2 (4.5) 1 (2.3)
Do you regret the implant treatment?	Yes No Missing	2 (6.1) 31 (93.9) 11 (−)
Would you do it again?	Yes Maybe Missing	31 (96.9) 1 (3.1) 12 (−)

### Study population

This prospective study involved partially edentulous patients lost their teeth in one or both jaws and treated under general anaesthesia with screw retained fixed implant bridges between 1 January 2006 to 31 December 2012 in a private clinic in Stockholm, Sweden. Informed consent was obtained from all individual participants included in the study. All treated patients had to be in a good general health condition to be eligible for general anaesthesia which was performed and monitored by an anaesthetist. The implant surgery itself did not differ from conventional implant procedure used for non-phobic patients treated without general anaesthesia.

### Inclusion criteria

Patients were selected according to the following inclusion criteria:

- Patients with dental phobia and severe pharyngeal reflexes.
- In good general health condition.
- With edentulous maxilla, mandible or both.
- With edentulous jaws minimum 6 months after extraction.
- With no bone augmentation prior or in combination with implant insertion.
- Implantation performed under general anaesthesia:
- With 4–6 Straumann implants (Straumann AG, Basel, Switzerland) in the maxilla.
- With 4–5 Straumann implants in the mandible.
- With screw retained fixed implant bridges.

### Exclusion criteria

Patients:

- Treated without general anaesthesia.
- Treated with other implant system than Straumann.
- With other rehabilitation than screw retained fixed implant bridges.
- Treated with bone augmentation were excluded.

### Treatment protocol

Patients were treated according to the following protocol:

1. Total extraction due to caries or periodontitis or both was done under general anaesthesia, followed by at least a 6-month healing period.
2. Interim removable dentures were produced in advance and used by the patient during the healing period.
3. Straumann implants were placed under general anaesthesia in the edentulous one jaw or in both (4–6 implants in maxilla, 4–5 implants in mandible).
4. A trans-gingival healing period of minimum 6 to 8 weeks before continuing the treatment (delayed loading).
5. Fixed implant bridge treatment was the final restoration.

### Protocol for general anaesthesia

Premedical evaluation of each patient was performed by the anaesthetist. Induction starts preoperatively in a peripheral venous line with 4 mg Betamethason, (Celestone, Merck & Co. Inc., Whitehouse Station, NJ USA), 0.5 mg Atropinesulphate (Myian AB, Stockholm, Sweden) and 2 g Bensylpenicillin (Meda AB, Solna, Sweden). In case of allergy to Bensylpenicillin, clindamycin was used (Clindamycin Orifarm, Stockholm Sweden). Fluid with glucose, Rehydrex 500 ml, was administered during anaesthesia (Fresenius Kabi, Halden Norway). Sedation with Propofol 1,5–2,5 mg/kg (Primex Pharmaceuticals, Helsinki, Finland) followed by Suxameton 25–50 mg (Celocurin Meda AB, Solna Sweden). An analgesic drug Fentanyl was used in doses of 20 microgram (Braun B, Melsungen AB, Germany) and repeated when needed. Intubation was done nasally with a silicon nasal tube, size 6 (Parker Medical, USA Colorado). Before start of surgery the patient is breathing unaided. Throughout the procedure the patient is monitored with ECG (QUIRUMED, Contec, Patient Monitor, Contec Medical Sustem CO LTD;Qinhuangdao, Hebi Province, China), saturation, blood pressure and CO_2_ production (Datex Ohmeda 5200 CO2 Capnography Anesterhesia Monitor, DRE Louiville, KY, USA).

### Protocol for surgical procedure under general anaesthesia

- Xylocain/adrenalin (Dentsply Pharmaceutical, ONY, United Kingdom) was used as local anaesthesia.
- Surgical flap was designed individually allowing good inspection of the bone and surrounding area.
- Straumann implants 4 to 6 and 4 to 5 were placed in the maxilla and mandible, respectively.
- The implants were inserted with external saline cooling of the drills.
- Healing abutments were applied for external healing.
- Wound closure was done with Vicryl 3–0 (Ethicon, Johnson & Johnson, Diegen, Belgium).
- The patients were allowed to use their soft relined removable dentures directly after implant insertion.
- A minimum of 6 to 8 weeks of healing time before impression taking for prosthetic restoration.

### Data collection

Data of the OHIP-14 questionnaire and the set of special questions were collected through follow-up visits at least 3 years after prosthetic treatment. The patients filled the patient consent and the questionnaires at the recall examination under supervision of one of the authors who is not involved in the treatment to avoid bias and any effects of interpersonal reactions. The individuals expressed satisfaction answering questions. The answers have score 0 to 5 where the scoring is distributed as following:

- (0) = never, (1) = hardly ever, (2) = occasionally, (3) = fairly often, (4) = very often, (5) = always. According to this scoring procedure low scores represent high patient satisfaction and better quality of life while higher scores gradually show less satisfied patients. As well as this study investigated the success of treatment with fixed full arched implant bridges in the maxilla, mandible or both. Treatment success was defined as functional dental implant bridges from 3 to 9 years after treatment.

### Data analysis/statistical methods

The Number of included patients, gender, age, number of installed implants and date of implant surgery were summarised using descriptive statistics, including mean, standard deviation (SD), median, range, frequency, and percentage.

Data of the OHIP-14 questionnaire and the set of special questions were descriptively summarized using descriptive statistics including frequencies, percentage, means and standard deviations as appropriate. Results are presented in tables (2-4) and graphs (1-4) for all patients. The OHIP-14 total score was analysed using logistic regression [36]. A dichotomization of the OHIP-14 total score into 0 vs. > 0 was used as response variable. The predictive ability of age, gender and location of implantation was tested by the use of the Wald chi-square test. Effect sizes were estimated as odds-ratios and associated 95% Wald confidence intervals. The functional success of the restored implant was recorded as either osseointegrated or failure (+/−).

## Results

Eighty two patients were treated with implants under general anesthesia between 01.01.2006 to 31.12.2012 and included and treated in this study. After start, 38 patients were excluded (4 died and 34 could not be reached to complete the follow-up after prosthetic treatment). One patient had missing data on several OHIP-14 items. The total patients’ number included in the analyses of the OHIP-14 was 43 (30–90 years). Table 2 shows the distribution of gender, age and location of implantation among these patients. The majority of patients were females (63.6%). 47.7% of the implants inserted in the maxilla and 31.8% of the patients had implants installed in both jaws.

**Table 2:** Background characteristics for included patients (*n* = 43)

Gender	Female, no. (%) Male, no. (%)	27 (63.6) 16 (36.4)
Age	Mean (SD) Min., Max Group 30–64 years, no (%) Group 65–90 years, no. (%)	62.8 (11.2) 32,90 20 (45.5) 23 (54.5)
Type of intervention	Maxilla, no. (%) Mandible, no. (%) Both, no. (%)	21 (47.7) 9 (20.4) 14 (31.8)

**Fig. 1:**
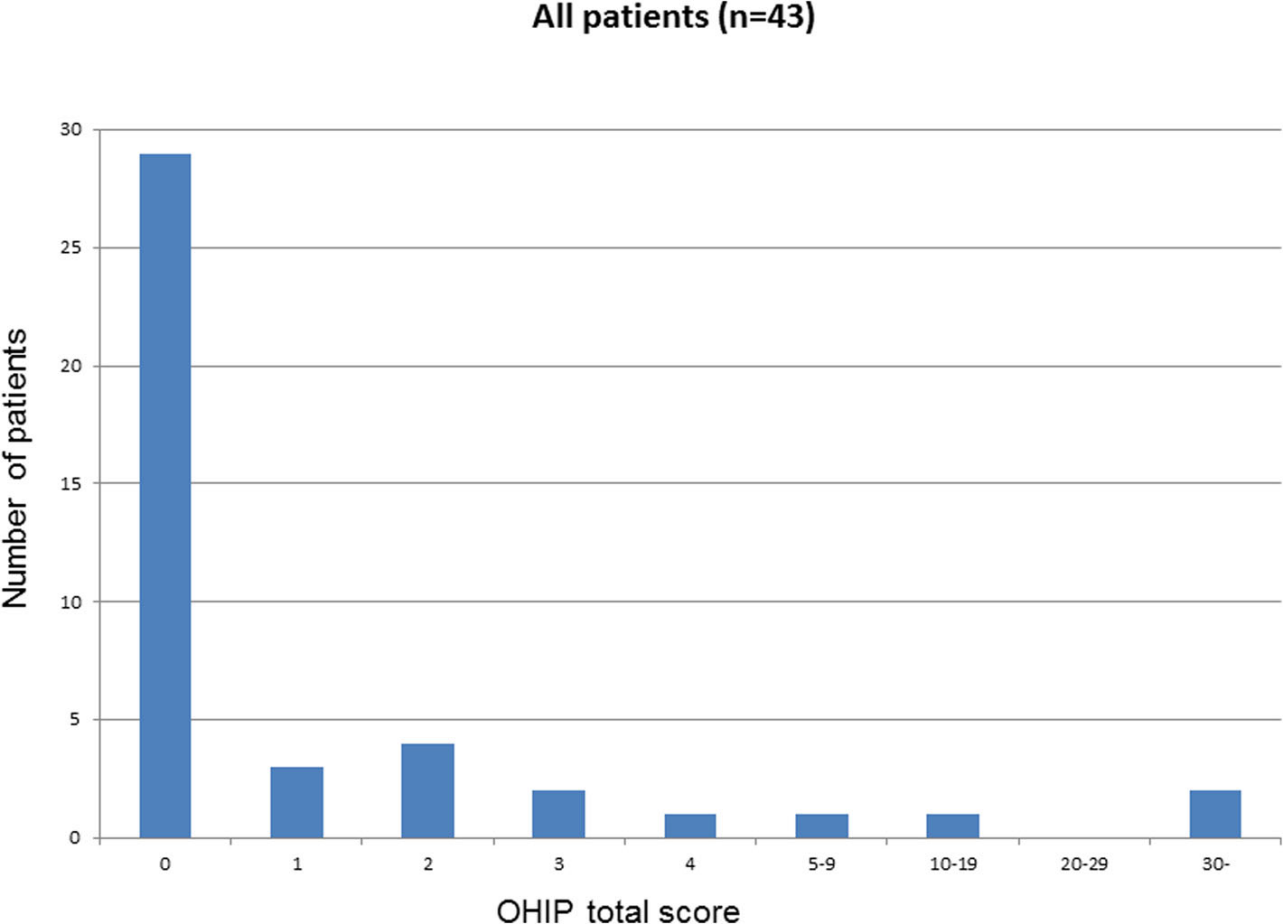
OHIP-14 total score distribution for all included patients. OHIP-14 total score is presented on the x-axis and number of patients on the y-axis

**Fig. 2:**
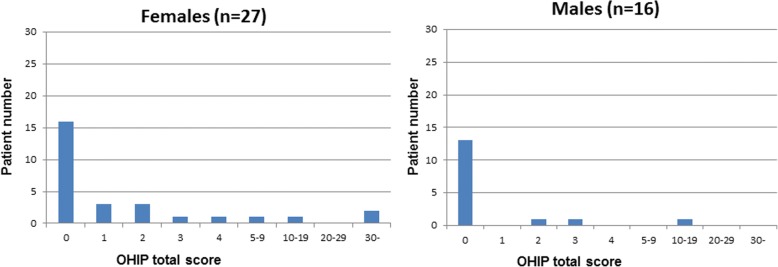
OHIP-14 total score distribution for female patients and male patients

**Fig. 3:**
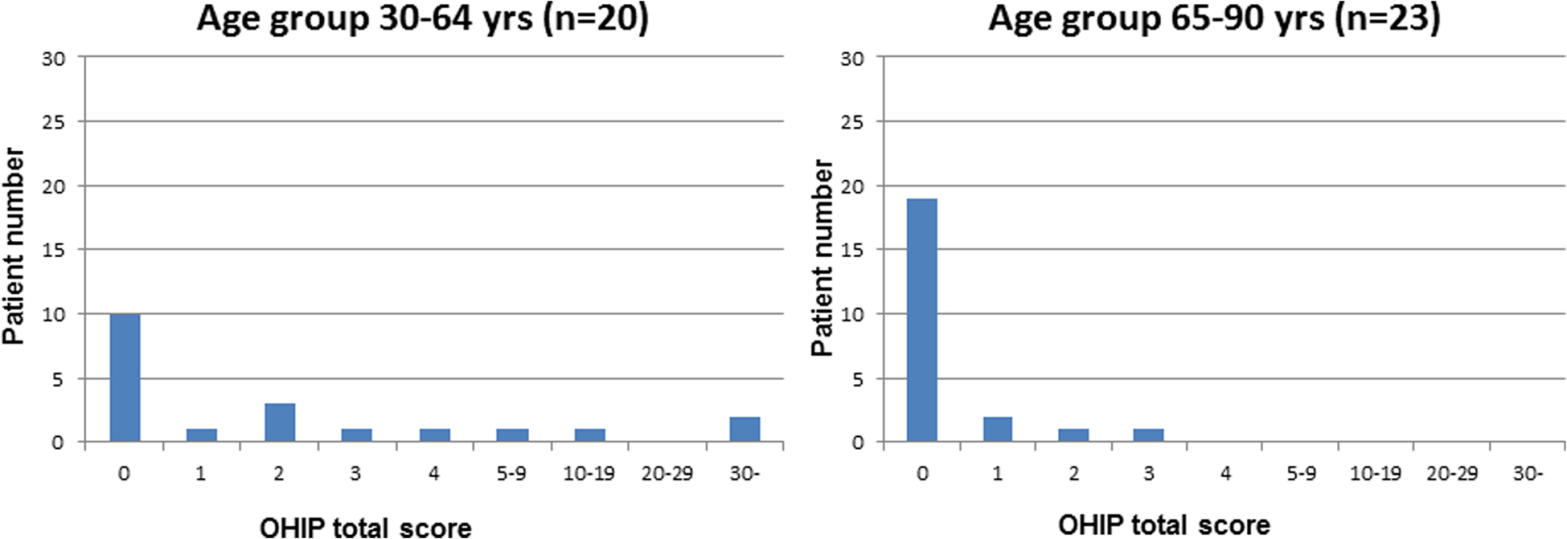
OHIP-14 total score distribution in age group (30-64) and in age group (65-90)

**Fig. 4:**
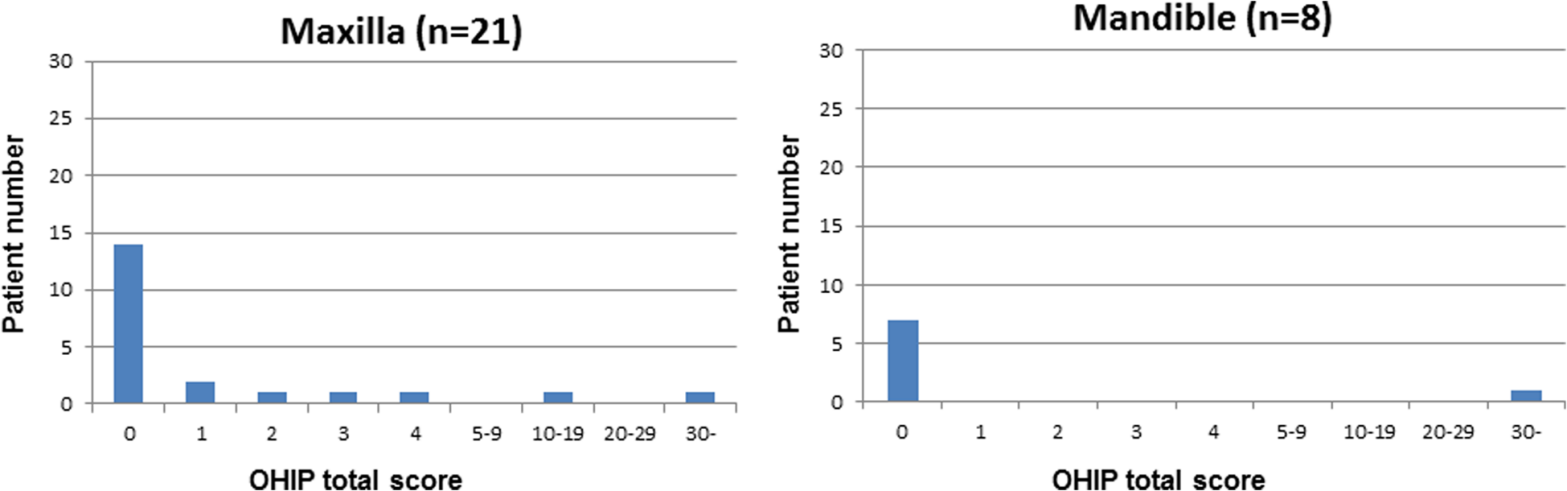
OHIP-14 total score distribution in maxilla and in mandible

The implant treatment of all 43 patients included in this study was successful as far as function and comfort. The follow-up period after the prosthetic reconstruction ranged from 3 to 9 years. Figure 1 shows the OHIP-14 total score distribution for all patients. The OHIP-14 total score was low for the majority of the patients with 67% scoring 0 and with a mean value total score of 2.5. The OHIP-14 total score by subgroups i.e. gender, age and type of intervention group are shown in Figs. 2, 3 and 4 respectively. The graphs seem to suggest some differences. However, the data indicate that younger patients (age group 30–64 years) especially young women are less satisfied (mean = 4.95+/− 9.81) than older patients (age group 65–90 years) with (mean = 0.3+/− 0.76). Logistic regression analysis (Table 3) was used to investigate the relationship between these background variables and the OHIP-14 total score.

**Table 3:** Results of logistic regression (*n* = 43). Odds-ratio estimates, associated confidence intervals and *p*-values

Effect	Estimated odds-ratio	95% confidence, limits	*P*-value
Gender	3.86	(0.68, 22.1)	0.129
Age	1.10	(1.02, 1.19)	0.014
Type of intervention:			
- both vs. maxilla	0.94	(0.19, 4.57)	0.608
- mandible vs. maxilla	3.49	(0.25, 48.14)	

## Discussion

The literature shows that patients satisfaction has been considered as an important criterion for treatment success since it is associated with compliance and in turn, anticipated treatment quality [9–11, 37]. In this study, the OHIP-14 questionnaire was administered to evaluate the level of satisfaction of patients suffer from dental phobia and SPRs with their implant treatment performed under general anesthesia. The first hypothesis of this study was confirmed because the results clearly demonstrated that the included patients are generally satisfied with their treatment and have good OHRQoL after treatment. The overall of patients have even changed their dental behaviour and continued after the performed oral rehabilitation to visit a dentist or oral hygienist for regular check-ups. The second hypothesis was confirmed in part because the obtained data showed that only age significantly affects patient satisfaction. Younger patients are less satisfied than older patients. But patients’ gender and location of implantation do not influence patient satisfaction. Evaluation of the results showed that the implant-supported bridges were successfully maintained in all patients after 3 to 9 years of function which confirm the third hypothesis. The success was measured as the retention of the original screw retained bridges over time. Similar results of success have been shown in several studies on patients treated without general anesthesia [9, 10, 37–39].

The OHIP-14 used in this investigation was previously validated and recommended for use in clinical studies [27, 28, 31]; it covers a wide range of oral health related problems, i.e. functional limitation, physical discomfort, psychological discomfort, physical disability, psychological disability, social disability and handicap [26, 29–31].

In this study, 43 out of 82 patients returned for regular check-ups which can be considered as a success because it is difficult for this category of patients to change their dental behaviour avoiding visiting a dental clinic. The reason for that could be that through their good satisfaction with their rehabilitation, patients realize the costly investment for the treatment and thus value the return of this investment in the form of sustaining good oral health.

Precise evaluation of the results indicates that only age has a statistically significant effect (*p* < 0.05) on patients’ satisfaction, reflecting that the number of patients viewing themselves as “problem free” increased with age. Analyses of data by subgroups indicate that younger patients especially women show more psychological discomfort and are less satisfied than older patients (Figs. 2 and 3). This is an interesting observation and may reflect that aesthetics has become an important issue in modern society [40] and that younger peoples’ social life style and attitude differ from older individuals’. These results are in line with a previous study [28] which also shows that oral discomfort has different influences on life depending on gender and age. Gender and location of the intervention showed in this study no significant influence on patients’ satisfaction (*P* > 0.05). However, a remarkable aspect is that, in all age groups presented in the graph 2, there are less satisfied women than men.

The results from the special questions showed that almost all patients (94.5%) are satisfied and (95.5%) would recommend the treatment to a friend or colleague.

These data are in accordance with Pjetursson et al. [41] finding; they find that more than 90% of patients treated with crowns or implant-supported fixed partial denture are completely satisfied. The obtained results confirm that 77.3% of the included patients in this study visited a dentist or oral hygienist for regular after treatment check-up. Most patients (93.9%) do not regret this kind of treatment and (96.6%) were willing to have the same treatment performed again if needed.

The findings of this study indicate that the preoperative psychological factors due to dental phobia and SPRs have no effect on post-treatment patients’ satisfaction with their implant treatment performed under general anesthesia.

From the results we conclude, with regard to the problem addressed that it is recommended to perform implant treatment on patients with dental phobia and SPR under general anesthesia. Consequently, implant-supported prosthesis would due to the availability of general anesthesia become a treatment option for these patients who otherwise would stay refusing any contact to the dental professionals who in turn have usually excluded implant treatment in cases involving patients with phobia or SPRs.

One question relevant to this topic is the impact of dental phobia from a social economic perspective. The comparably high cost for implant treatment under general anesthesia versus removable dentures could be the major reason for primary limitation for choosing this therapy. However, if more patients were able to choose this therapy it could in the long run reduce other costly health care consumption such as depression treatment and medication. This is a discussion worth pursuing in further studies.

Various investigations were made to study satisfaction with implant treatment [41, 42] But to the knowledge of the authors of this study, there is no study investigated satisfaction of patients suffering from dental phobia or SPR after implant treatment under general anesthesia. Therefore, this study fills an important gap in the academic field and should be used to promote a debate.

## Conclusion

Based on this study results, it is assumed that treatment using implants is feasible for patients suffering from dental phobia and SPRs. Therefore, these patients can be offered the same implant treatment options as non-phobic patients, with similar success rates.

## Annex

### OHIP-14 the Oral Health Impact Profile

Dimension Question

Functional

1. Have you had trouble pronouncing any words because of problems limitation with your teeth, mouth or dentures?

2. Have you felt that your sense of taste has worsened because of problems with your teeth, mouth or dentures?

Physical pain

3. Have you had painful aching in your mouth?

4. Have you found it uncomfortable to eat any foods because of problems with your teeth, mouth or dentures?

Psychological discomfort

5. Have you been self-conscious because of your teeth, mouth or discomfort dentures?

6. Have you felt tense because of problems with your teeth, mouth or dentures? Physical disability.

7. Has your diet been unsatisfactory because of problems with your teeth, disability mouth or dentures?

8. Have you had to interrupt meals because of problems with your teeth, mouth or dentures?

Psychological disability

9. Have you found it difficult to relax because of problems with your disability teeth, mouth or dentures?

10. Have you been a bit embarrassed because of problems with your teeth, mouth or dentures?

Social disability

11. Have you been a bit irritable with other people because of problems disability with your teeth, mouth or dentures?

12. Have you had difficulty doing your usual jobs because of problems with your teeth, mouth or dentures? Handicap

13. Have you felt that life in general was less satisfying because of problems with your teeth, mouth or dentures?

14. Have you been totally unable to function because of problems with your teeth, mouth or dentures?

## Abbreviations

OHIP: Oral health impact profile
OHRQoL: Oral health quality of life
SD: Standard deviation
SPR: Severe pharyngeal reflexes
